# Work-related acute physical injuries, chronic overuse complaints, and the psychosocial work environment in Danish primary care chiropractic practice – a cross-sectional study

**DOI:** 10.1186/s12998-018-0174-2

**Published:** 2018-02-13

**Authors:** Mille Charlotte Hansen, Tine Aagaard, Henrik Wulff Christensen, Jan Hartvigsen

**Affiliations:** 1 Private practice of chiropractic, Ballerup, Denmark; 2 Department of Sports Science and Clinical Biomechanics, University of Southern Denmark, Campusvej 55, DK-5230 Odense M, Denmark; 3 Nordic Institute of Chiropractic and Clinical Biomechanics, Campusvej 55, DK-5230 Odense M, Denmark

**Keywords:** Chiropractic, Work-related, Injuries, Psychosocial, Stress, Overuse, Complaints

## Abstract

**Background:**

Little is known about the physical and psychosocial work environment of chiropractors and their work-related health complaints, and this has never been described for Danish chiropractors. The aim of this study was, therefore, to describe work-related acute physical injuries, overuse complaints, and psychosocial stress in Danish chiropractic work settings.

**Methods:**

We developed a questionnaire specifically for this study and distributed it electronically in August 2016 using SurveyXact to all 575 members of the Danish Chiropractors’ Association working in primary care clinics. Chiropractors were asked about their work-related acute physical injuries and overuse complaints as well as any psychosocial stress they experienced at work during the previous year. We described our sample and variables using means, medians, ranges, and confidence intervals where appropriate. Statistically significant differences between genders, types of complaints and injuries, and between clinic owners and associates were examined using Chi-square and Fischer’s exact tests, where appropriate, or by examining confidence intervals for non-overlap.

**Results:**

355 (65.2%) chiropractors answered the survey. Of these, 216 (61%, 95% CI 56–66) had experienced a work-related acute physical injury and/or overuse complaint during the previous year. Work-related overuse complaints were most commonly reported in the low back, wrist, thumb, and shoulder, and were more common among women (63%, 95% CI 56–70) than men (51%, 95% CI 43–59). Chiropractors with more than five years in practice (59%, 95% CI 52–64) reported significantly fewer work-related acute injuries and overuse complaints during the previous year compared with chiropractors with less than five years in practice (83%, 95% CI 73–91). In general, these practicing Danish chiropractors reported having a good psychosocial work environment, and 90% of chiropractors “always” or “often” felt that they were motivated and committed to their work.

**Conclusion:**

This sample of Danish practicing chiropractors commonly reported work-related acute physical injuries or overuse complaints. Overuse complaints were most commonly reported in the low back, wrist, thumb, and shoulder and were more common among women than men. Newly educated chiropractors reported more overuse complaints than experienced chiropractors. Collectively, this sample of Danish chiropractors reported that they had a good psychosocial work environment.

## Background

A work-related injury is defined as an injury that occurs as a result of a work-related activity [1, 2]. An acute injury happens as a consequence of an excessive peak load, and an overuse injury develops over a prolonged period due to repetitive physical loading of a tissue [3].

Mental health is also affected by the work environment through psychosocial exposures. Physical and mental exposures at work are, however, not the only cause of physical and mental health problems because physical exposures during leisure time, i.e., doing sports and physical activity, and problems, for example in the family, can contribute to accumulated physical and mental load [4]. Individuals differ, so even for those who are exposed to the same type of loads at work, their gender, age, and general physical and mental resilience play an important role in their predisposition to developing work-related health problems [5].

Chiropractic in Denmark has become a university-trained profession, and approximately half of all chiropractors now have a Master’s degree from the University of Southern Denmark [6]. Today, 579 Danish chiropractors are active in the labour market, and only 30 of these do not work in primary care practice in Denmark. Instead, they are working in secondary care at hospitals, or they are employed as full-time teachers or researchers [7]. As part of their work, practicing chiropractors use different manual techniques and exercises that typically expose them to repeated lifting, bending, twisting, and reaching while performing their work [8]. Such loads increase the risk of developing low back pain as well as the risk of developing pain and injuries in the shoulders, wrists, and thumbs [9, 10].

Few studies have investigated the prevalence of work-related injuries or complaints among chiropractors. Mior et al. found that among Canadian chiropractors, 87% reported back pain. Men more frequently reported low back pain (59%), whereas women had a higher prevalence of thoracic pain (79%) [11].

Holm et al. found that out of 397 chiropractors in the United States, 40% had experienced at least one acute work-related injury during their career as a chiropractor [10]. The most commonly affected body parts were wrist, hand, finger (42.9%), shoulder (25.8%) and low back (24.6%). Most injuries were soft tissue injuries, and 67% occurred while performing manipulation of the patient. Interestingly, 37% of the injuries happened during the first five years of working as a chiropractor [10]. Finally, in a study of chiropractors in the United Kingdom published in 2008, 51.7% reported having had a work-related injury during their career as a chiropractor, most frequently in the shoulder (28%) followed by the low back (23%), and again the majority (two out of three) reported that the injury had occurred during the first five years after graduation [12].

Other healthcare professions such as physical therapists perform many daily tasks that are similar to those of chiropractors. Vieira et al. reviewed 32 studies dealing with work-related musculoskeletal disorders among physical therapists and found that between 53 and 91% of physical therapists experienced work-related musculoskeletal disorders during their worklife [13]. The most commonly affected body part across studies was the low back; female therapists reported more symptoms than male; and therapists working in hospitals and engaged in patient transfer, a task rarely performed by chiropractors, was commonly associated with symptoms in the back, neck, and shoulders. Manual therapy and treating many patients in a day were associated with pain and symptoms in the thumb and hands [13].

### Aim

The aim of this study was to describe work-related physical acute injuries and overuse complaints as well as psychosocial stress among chiropractors in primary care chiropractic practice in Denmark during the previous year.

## Methods

### Study design

A descriptive cross-sectional study was undertaken.

### Participants

All chiropractors practicing in primary care chiropractic clinics in Denmark and who were members of the Danish Chiropractors’ Association (DCA) were invited to participate (*n* = 575). The exact number of chiropractors in Denmark without membership of DCA is not known, but estimated >95% of chiropractors in Denmark are members of the DCA. For chiropractors in Denmark, it is not required to be a member.

### Setting and procedures

We designed the questionnaire used in this survey by identifying relevant domains based on surveys used previously by the Danish National Research Centre for the Working Environment, and we subsequently populated the domains with specific questions from these surveys [14]. In addition, we took relevant questions from other surveys on work-related injuries in chiropractors (Table 1). The questionnaire was delivered electronically via SurveyXact (Aarhus, Denmark) and potential participants were invited via email to participate by clicking on a person-unique link provided in the email. Care was taken to minimize the time it took to fill in the questionnaire by including skipping functions wherever feasible so that participants only answered questions related to their complaints. It was possible to pause in the filling out of the questionnaire and return to completing the remaining parts at a later time. All questions pertained to physical and mental complaints during the previous year. We initially tested the feasibility and face-validity of the questionnaire by asking four chiropractors to fill in the electronic questionnaire and provide feedback. We subsequently performed minor modifications and clarifications. The questionnaire was sent out in August 2016. We sent reminders to potential participants after 7 and 14 days, if they had only filled out part of the questionnaire or had not responded at all. After 19 days, the data collection was closed, after which the data analysis was started. Data collection was administered by a research assistant from the Nordic Institute of Chiropractic and Clinical Biomechanics, where data were also stored on a secure server.

**Table 1:** Domains and items in the electronic questionnaire

Domain	Descriptions
1	Demographic data: Questions about gender, age, graduation, conditions of employment, and working hours with patient contact [6].
2	Working positions when treating patients in carrying out manual work on a daily basis.
3	Work-related acute injuries and overuse complaints during the previous year and detailed questions for every body part if these injuries or complaints were currently present [10].
4	Psychosocial work environment and questions about inability to work because of mental health problems [14].
5	Chronic disease that affects work as a chiropractor in primary care.
6	Consequences of work-related physical and/or mental issues [10].

### Analyses

We described our sample and variables using means, medians, ranges, and confidence intervals where appropriate. Statistically significant differences between genders, types of complaints and injuries, and between clinic owners and associates were examined using Chi-square and Fischer’s exact tests, where appropriate, or by examining confidence intervals for non-overlap. Statistical significance was set at *P* < 0.05. Analysis was performed in STATA version 14 (College Station, TX, USA) and in Microsoft® Excel 2011 (Redmond, WA, USA).

### Ethics

This survey was conducted through the Danish Chiropractors’ Association. All responses were anonymous and individual chiropractors or their clinics could not be identified by the researchers. According to Danish law, anonymous surveys protect the anonymity of the population and therefore do not require ethical approval.

## Results

**Fig. 1:**
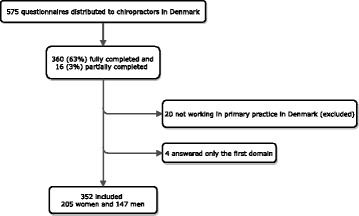
Flowchart of the inclusion and exclusion of the respondents and ongoing deleted respondents. One participant only completed the first question

Three hundred and seventy-six of the 575 invited chiropractors (65.2%) answered the electronic questionnaire and 356 of these (94.7%) worked in primary care chiropractic practice, with 207 (58.3%) being women (Fig. 1). Participants were on average 43 years old and had practiced as chiropractors for 15 years. Sixty-two per cent were clinic owners and 37% were employed as associates. On average, women worked 28 h/week with patient contact, whereas men saw patients on average 32 h/week (Table 2).

**Table 2:** Descriptive data from chiropractors in primary care practice in Denmark, *n* = 355

	Women	Men	Total
Gender n (%)	207 (58,3)	148 (41,7)	355 (100)
Age group in years n (%)			
- 21–30	36 (17,4)	18 (12,2)	54 (15,2)
- 31–40	54 (26,1)	38 (25,7)	92 (25,9)
- 41–50	64 (30,9)	39 (26,4)	103 (29,0)
- 51–60	44 (21,3)	35 (23,6)	79 (22,3)
- 61–70	9 (4,3)	17 (11,5)	26 (7,3)
- >70	0 (0)	1 (0,6)	1 (0,3)
Years since graduation n (%)			
- 0–5	47 (22,7)	29 (19,6)	76 (21,4)
- 6–10	33 (15,9)	21 (14,2)	54 (15,2)
- 11–15	37 (17,9)	20 (13,5)	57 (16,0)
- 16–20	14 (6,8)	10 (6,8)	24 (6,8)
- >21	76 (36,7)	68 (45,9)	144 (40,6)
Employment (more than one answer possible n (%)			
- Owner of a clinic	118 (57,0)	102 (68,9)	220 (62,0)
- Associate	86 (41,5)	44 (29,7)	130 (36,6)
- Internship	14 (6,8)	8 (5,4)	22 (6,2)
- Teacher	9 (4,3)	11 (7,4)	20 (5,6)
- Researcher	4 (1,9)	4 (2,7)	8 (2,3)
- Employed in the public sector	8 (3,9)	5 (3,4)	13 (3,7)
- Employed in health care insurance	14 (6,8)	10 (6,8)	24 (6,8)
- Other	9 (4,3)	11 (7,4)	20 (5,6)
Region n (%)			
- North Denmark	19 (9,3)	9 (6,1)	28 (7,9)
- Central Denmark	49 (23,9)	33 (22,3)	82 (23,2)
- Southern Denmark	55 (26,8)	44 (29,7)	99 (28,1)
- Zealand	31 (15,1)	19 (12,8)	50 (14,2)
- Capital	51 (24,9)	43 (29,1)	94 (26,6)
*Missing*	2	0	2
Country of graduation n (%)			
- Denmark	119 (57,5)	73 (49,3)	192 (54,1)
- United Kingdom	41 (19,8)	31 (21,0)	72 (20,3)
- USA	44 (21,3)	41 (27,7)	85 (23,9)
- Canada	3 (1,4)	3 (2,0)	6 (1,7)
- Other country	0 (0)	0 (0)	0 (0)
*Missing*	2	0	2
Self-reported hypermobility n (%)			
- Yes	44 (21,3)	3 (2,0)*	47 (13,3)
- No	160 (77,3)	142 (96,6)	302 (85,3)
- Don’t know	3 (1,4)	2 (1,4)	5 (1,4)
*Missing*	0	1	1
Hours with patient contact and treatment in primary practice pr. week. n (%)*			
- ≤10	6 (2,9)	2 (1,3)	8 (2,3)
- 11–20	16 (7,8)	5 (3,4)	21 (6,0)
- 21–25	30 (14,6)	11 (7,5)	41 (11,6)
- 26–30	60 (29,3)	31 (21,1)	91 (25,9)
- 31–35	67 (32,7)	62 (42,2)	129 (36,6)
- 36–40	20 (9,8)	26 (17,7)	46 (13,1)
- >40	6 (2,9)	10 (6,8)	16 (4,5)
*Missing*	2	1	3
Follow-up consultations at 1 h if only follow-ups. N (%)			
- <2	14 (6,9)	9 (6,1)*	23 (6,5)
- 2–4	96 (47,0)	49 (33,3)	145 (41,3)
- 5–6	88 (43,1)	72 (49,0)	160 (45,6)
- 7–8	4 (2,0)	11 (7,5)	15 (4,3)
- >8	2 (1,0)	6 (4,1)	8 (2,3)
*Missing*	3	1	4
New patients incl. Patients with relapse over one year at an average per day n (%)			
- 0–1	32 (15,7)	13 (8,9)	45 (12,8)
- 2–4	150 (73,5)	119 (81,5)	269 (76,9)
- 5–7	20 (9,8)	10 (6,9)	30 (8,6)
- >7	2 (1,0)	4 (2,7)	6 (1,7)
*Missing*	3	2	5

Percentage indicated in parentheses, and figured out the total number of women or men

*statistically significant difference between men and women with *P* < 0,05

^a^The possibility to give more than one answer was explicitly stated in the questionnaire

**Fig. 2:**
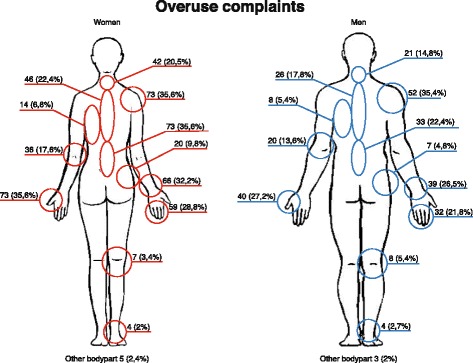
Localization of work-related overuse complaints of chiropractors in Denmark during the previous year, *n* = 204. 204 chiropractors in primary care practice reported overuse complaints and these occurred in 129 women (62%) and 75 men (51%). In the questionnaire, it was possible to choose complaints or pain in more than one body part. The percentage is calculated from all included respondents who answered the questionnaire, reported by gender

Two hundred and fourteen of the 352 (60.8%) had experienced a work-related acute physical injury (19 (5.4%)) and/or overuse complaints (204 (58.0%)) during the previous year with nine (2.6%) indicating that they had experienced both (Figs. 2 and 4).

### Overuse complaints

Work-related overuse complaints during the previous year were most commonly reported in the low back, wrist, thumb, and shoulder, and were more common among women (62%, 95% CI 56–70) than men (51%, 95% CI 43–59) (Fig. 2).

**Fig. 3:**
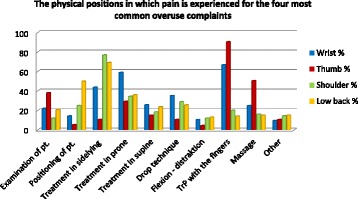
The physical positions in which pain is experienced for the four most common overuse complaints, n = 204. Situations in which pain occurs in the case of overuse complaints of chiropractors in primary care practice in Denmark during the previous year. Shown in percentages because the prevalence of overuse pain in each body part was not the same: Wrist (*n* = 105); Thumb (*n* = 113); Shoulder (*n* = 125); and Low back (*n* = 106). It was possible to choose more than one answer. pt. = patient. TrP = triggerpoint treatment

When asked about the situation in which pain occurs, in the case of overuse complaints, most chiropractors indicated that manual treatment of myofascial trigger points caused pain in the thumb (90.3%) and wrist (66.7%). Fifty-nine per cent also reported pain in the wrist when doing manipulation treatment with the patient in the prone position. Shoulder pain (76.8%) and low back pain (68.9%) were most frequently associated with manipulating the spine of patients in side-lying position (Fig. 3).

Of those reporting overuse complaints in one of the four most common body parts (low back, wrist, thumb, and shoulder), 50% of chiropractors reported mostly receiving treatment from colleagues in the clinics. Less than 5% received treatment in the secondary care sector, and less than 7% used analgesics as the primary treatment for their pain.

When looking at treatment positions, chiropractors who often treated patients’ low back in a side-lying position reported pain in the shoulder (34%) and the low back (30%).

### Acute injuries

Work-related acute physical injuries during the previous year were uncommon and similar between women (6% (95% CI 3–11)) and men (4% (95% CI 2–9)).

**Fig. 4:**
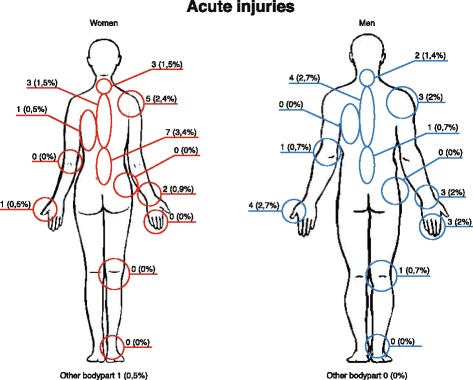
Localization of work-related acute injuries in chiropractors in Denmark during the previous year, *n* = 19. 19 chiropractors in primary care practice reported acute injuries and these occurred in 13 women (6%) and six men (4%). In the questionnaire, it was possible to choose pain in more than one body part. The percentage is calculated from all included respondents who answered the questionnaire, reported by gender

The low back (3.4% women; 0.7% men) was the most commonly injured site followed by the shoulder (2.4% women; 2.0% men), and midback (1.5% women; 2.7% men) (Fig. 4). Most acute injuries had occurred when treating patients in a side-lying position doing manipulation or mobilization treatment of the low back or pelvis.

Chiropractors with more than five years in practice (59%, 95% CI 52–64) reported fewer work-related acute injuries and overuse complaints during the previous year compared with chiropractors with less than five years in practice (83%, 95% CI 73–91).

A work-related acute injury or overuse complaint during the previous year, was reported by 67% of chiropractors who saw patients 26–30 h per week, 56% of chiropractors who saw patients 31–35 h per week, and 43% of chiropractors who saw patients >40 h per week.

Slightly more clinic owners (15% (95% CI 11–20)) than associates in primary care practice (8% (95% CI 4–14)) had been unable to work because of acute physical injuries or overuse complaints during the previous year.

### Psychosocial work environment

Generally, practicing Danish chiropractors reported that they had a good psychosocial work environment. Fifty-seven percent of clinic owners and 55% of associates indicated that they “rarely” or “never” experienced emotionally stressful situations because of their work. Almost 90% (88%) of clinic owners felt “always” or “often” that problems at work were also their personal problems compared with around 40% (38%) of associates. More than 60% of the respondents indicated that they “rarely” or “never” expressed their opinion on work-related psychosocial issues (Table 3).

**Table 3:** The psychosocial work environment of chiropractic clinic owners and associates in primary care practice in Denmark, *n* = 340

Psychosocial work environment	Always n(%)	Often n(%)	Sometimes n(%)	Rarely n(%)	Never n(%)
Does your work put you in emotionally stressful situations?*					
- Clinic owner	0 (0)	12 (5,5)	81 (37,5)	76 (35,2)	47(21,8)
- Associate	1 (0,8)	8 (6,5)	47 (37,9)	57 (45,9)	11 (8,9)
Does your work demand that you do not state your opinion?					
- Clinic owner	0 (0)	12 (5,5)	76 (35,2)	73 (33,8)	55 (25,5)
- Associate	0 (0)	6 (4,8)	37 (29,8)	41 (33,1)	40 (32,3)
Is your workload unevenly distributed causing your tasks to pile up?					
- Clinic owner	3 (1,4)	13 (6,0)	85 (39,4)	82 (37,9)	33 (15,3)
- Associate	0 (0)	8 (6,5)	42 (33,8)	61 (49,2)	13 (10,5)
How often are you delayed compared to your planned appointments?					
- Clinic owner	1 (0,5)	39 (18,1)	69 (31,9)	84 (38,9)	23 (10,6)
- Associate	0 (0)	19 (15,3)	51 (41,1)	43 (34,7)	11 (8,9)
How often does it happen that you do not get all your tasks done?					
- Clinic owner	1 (0,5)	23 (10,6)	55 (25,5)	102 (47,2)	35 (16,2)
- Associate	0 (0)	5 (4,0)	32 (25,8)	54 (43,6)	33 (26,6)
Have you influence over the amount of work?*					
- Clinic owner	118 (54,6)	73 (33,8)	21 (9,7)	3 (1,4)	1 (0,5)
- Associate	36 (29,1)	61 (49,2)	19 (15,3)	7 (5,6)	1 (0,8)
How often do you look for work elsewhere?*					
- Clinic owner	3 (1,4)	9 (4,2)	13 (6,0)	27 (12,5)	164 (75,9)
- Associate	2 (1,6)	7 (5,6)	15 (12,1)	37 (29,9)	63 (50,8)
Do you get information at work about important decisions, changes,and future plans in good time?*					
- Clinic owner	170 (78,7)	30 (13,9)	12 (5,5)	1 (0,5)	3 (1,4)
- Associate	35 (28,2)	45 (36,3)	27 (21,8)	15 (12,1)	2 (1,6)
Do you feel motivated and committed to your work?*					
- Clinic owner	144 (66,6)	62 (28,7)	9 (4,2)	1 (0,5)	0 (0)
- Associate	59 (47,6)	55 (44,3)	9 (7,3)	1 (0,8)	0 (0)
Do you feel that the workplace problems are also yours?*					
- Clinic owner	152 (70,4)	38 (17,6)	17 (7,8)	8 (3,7)	1 (0,5)
- Associate	11 (8,9)	36 (29,1)	44 (35,5)	26 (20,9)	7 (5,6)

216 clinic owners, 124 associates. 12 people with other types of employee status are not included in this table

*Statistically significant difference between clinic owners and associates with *P* < 0.05

Sixty percent of associates compared with 53% of all clinic owners indicated that they “rarely” or “never” experienced that their workload was unevenly distributed causing their tasks to pile up. More than 50% of both clinic owners and associates indicated that they were “often” or “sometimes” behind schedule during a normal work day. Only about 10% of clinic owners and 4% of associates indicated that they “often” did not get all their tasks done. Almost 80% of clinic owners and associates reported that they “always” or “often” had influence over their workload. On the slightly negative side, 34% of associates indicated that they “sometimes” or “rarely” got timely information at work about important decisions affecting their work compared with 6% of clinic owners. Importantly, 90% of all clinic owners as well as 90% of all associates “always” or “often” felt that they were motivated and committed to their work. Consequently, 80% of all associates and almost 90% of all clinic owners “rarely” or “never” looked for work elsewhere (Table 3).

### Unable to work because of health problems

A similar proportion of clinic owners (10% (95% CI 6–14)) and associates (12% (95% CI 7–19)) had been unable to work because of mental health problems during the previous year.

For those who had been unable to work because of mental health problems (*n* = 36), 75% had talked about these problems with colleagues, approximately 30% (*n* = 11) had consulted a psychologist, and approximately 30% did meditation or mindfulness regularly.

As a consequence of physical acute injuries and/or overuse complaints and mental health problems (*n* = 217), 75% had changed their work position, about 66% had modified their technique, and about 33% had decreased their working hours.

Nine participants indicated that their current pain was connected to a previous lumbar disc prolapse. Seven indicated that they had a previous shoulder injury or overload pain which still gave them pain.

Some (*n* = 13) of the chiropractors had chronic diseases or complaints including four with a chronic disease of the gastrointestinal tract and three with symptoms after a stressful period. These events significantly influenced their work as a chiropractor.

## Discussion

Danish practicing chiropractors commonly reported physical work-related acute injuries or overuse complaints. Overuse complaints were significantly most commonly reported in the low back, wrist, thumb, and shoulder, and were more common among women than men. Chiropractors with less than five years in practice reported more overuse complaints than chiropractors with more than five years in practice. They generally reported a good psychosocial work environment and rarely thought about changing jobs.

Previous research has investigated work-related injuries among chiropractors from a career perspective. Holm and Rose found that 40% of chiropractors in their American survey reported an injury during their career and Acott-Smith reported a work-related injury in 51.7% of British chiropractors during their career [10, 12]. We looked at the one-year prevalence of both acute injuries and overuse symptoms, and we found that shoulder pain and low back pain were commonly experienced in chiropractors who treat patients in a side-lying position, where they stand with their trunk in a flexed and rotated position, a similar finding to that reported in both the Holm and Rose and Acott-Smith studies [10, 12]. Both studies found that most injuries occured early on in the chiropractor’s career, which is similar to our findings among Danish chiropractors [10, 12]. Also among physical therapists, less experienced clinicians faced more work-related musculoskeltal symptons with at least 45% of participants across studies reporting their first complaint within their first five years of practice [13]. Mior and Diakow surveyed Canadian chiropractors in 1987 and found a higher prevalence of low back pain in men, whereas in our study, women reported more pain in the low back [11]. Among physical therapists, women report more musculoskeletal complaints than men across studies including back, neck, wrist, and hand symptoms [13].

The psychosocial work environment of young Danish medical doctors was studied by Varma and Bonde and they found that 20% of the young doctors, who commonly work in hospitals, experienced “high” or “very high” emotional stress in their work [15]. Among chiropractors, the corresponding number was only 6% for both clinic owners and associates. In addition, 5% of the young medical doctors indicated that they had “low” or “very low” motivation and commitment to their work, whereas the corresponding number for the chiropractors in our survey was less than 1% [15]. In the study by Holm and Rose, 69.8% of the respondents reported that they did not need to take any time off work as a result of their injury [10]. Acott-Smith found that 76.9% did not take time off work. This is similar to the results from our study where 77.3% had not been unable to work because of their injury or overuse complaints [12].

We believe that our study has several strengths and paints a realistic picture of physical and mental work-related complaints among Danish chiropractors. First, it was distributed to all chiropractors in Denmark who were members of DCA, and we had a reasonable response rate (66% of all members). Due to the confidential nature of the survey, we were not able to ascertain information about non-responders and we were also unable to obtain information from chiropractors who may have left private practice for health reasons.

We also chose to focus on chiropractors in primary care clinics because this is where the large majority of chiropractors in Denmark work. Finally, we based our questions in surveys that had been used previously in other occupational groups in Denmark, and we tested the questionnaire for feasibility.

Weaknesses include the likelihood that chiropractors who had experienced work-related health problems were more inclined to fill in the questionnaire and for those who did not, they may have felt that information about work-related health problems was a private matter. Another limitation is that it was open for participants to interpret the terms “acute injuries” and “overuse complaints” in the questionnaire. It could have lead to a minor misclassification, probably in the direction of overuse complaints because of the variable importance and the highest number of answers. Finally, the questionnaire was very long, and the survey completion time was 5–30 min depend on the number of complaints, which may have caused some to give up. In retrospect, we could probably have omitted some of the questions relating to the finer details that we ended up not including in this paper. Also, we did not probe into the interplay between work and leisure time exposures.

The practical implication of this knowledge is that chiropractors could probably prevent a significant number of work-related complaints by focusing on the biomechanics of their working postures, in particular when performing sideposture manipulation, in order to minimize twisting and bending. Further, chiropractors should limit the use of manual treatment of soft-tissues in order to minimize the stress to thumbs and fingers. Because of the high prevalence of injuries and complaints among recently graduated chiropractors found in this and other studies, educational programs should focus on work positions and body mechanics, and experienced clinical supervisors and teachers should focus on giving feedback on work positions. Future research should focus on evaluating preventive measures as well as on longitudinal studies of cohorts of chiropractors, where determinants of longer lasting problems and chronicity can be identified.

## Conclusions

Danish practicing chiropractors commonly reported physical work-related acute injuries or overuse complaints. Overuse complaints are significantly more common in women and occur primarily in the low back, wrist, thumb, and shoulder. Chiropractors with less than five years in practice report more overuse complaints than chiropractors with more than five years in practice. Chiropractors in Denmark generally have a good psychosocial work environment. The need for further research is important and relevant preventive strategies in chiropractic education must be a high priority.

## References

[1] Holder NL, Clark HA, DiBlasio JM, Hughes CL, Scherpf JW, Harding L, Shepard KF (1999). Cause, prevalence, and response to occupational musculoskeletal injuries reported by physical therapists and physical therapist assistants. Phys Ther.

[2] Brock W, Norwood J (1987). Occupational Injuries and Illnesses in the United States by Industry, 1985.

[3] Luttmann A, Jäger M, Griefahn B, Caffier G, Liebers F, Steinberg U. Preventing musculoskeletal disorders in the workplace. In: Protecting Workers' Health series, vol. 5. Geneva: World Health Organization; 2003.

[4] Roos E, Bliddal H, Christensen R, Hartvigsen J, Mølgaard C, Søgaard K, Zebis M (2013). Forebyggelse af skader og sygdomme i muskler og led. København: Vidensråd for forebyggelse.

[5] Andersen JH, Haahr JP, Frost P (2007). Risk factors for more severe regional musculoskeletal symptoms: a two-year prospective study of a general working population. Arthritis Rheum.

[6] Nielsen OL, Kongsted A, Christensen HW (2015). The chiropractic profession in Denmark 2010-2014: a descriptive report. Chiropractic & manual therapies.

[7] Østergaard J: Dansk Kiropraktor Forenings medlemstal pr. 1. januar 2017. In*.* København: Dansk Kiropraktor Forening; 2017.

[8] Ndetan HT, Rupert RL, Bae S, Singh KP (2009). Epidemiology of musculoskeletal injuries among students entering a chiropractic college. J Manip Physiol Ther.

[9] Frymoyer JW, Pope MH, Costanza MC, Rosen JC, Goggin JE, Wilder DG (1980). Epidemiologic studies of low-back pain. Spine.

[10] Holm SM, Rose KA (2006). Work-related injuries of doctors of chiropractic in the United States. J Manip Physiol Ther.

[11] Mior SA, Diakow PR (1987). Prevalence of back pain in chiropractors. J Manip Physiol Ther.

[12] Acott-Smith G (2008). Occupational Musculoskeletal Injuries of Chiropractors in the UK: A Survey.

[13] Vieira E, Schneider P, Guidera C, Gadotti I, Brunt D (2016). Work-related musculoskeletal disorders among physical therapists: a systematic review. J Back Musculoskelet Rehabil.

[14] Arbejdsmiljøinstitutet (2005). Arbejde i ældreplejen - Spørgeskema til medarbejdere og ledere i ældreplejen i danske kommuner.

[15] Varma A, Bonde J (2012). Yngre lægers arbejdsvilkår 2012 - en spørgeskemaundersøgelse af medlemmer i Foreningen af Yngre Læger.

